# The roles and experiences of informal caregivers in non-malignant respiratory disease at the end of life: A thematic synthesis of qualitative studies

**DOI:** 10.1017/S1478951526101643

**Published:** 2026-02-13

**Authors:** Kathy Rogers, Candy McCabe, Natasha Bradley, Alison Llewellyn

**Affiliations:** 1 Centre for Health and Clinical Research, School of Health and Social Wellbeing, College of Health, Science and Society, University of the West of England, Bristol, UK; 2 Centre for Education and Research, Dorothy House Hospice Care, Winsley, UK; 3 Marie Curie Palliative Care Research Department, Division of Psychiatry, University College London and Research, Policy & Public Affairs Directorate, Marie Curie, One Embassy Gardens, London, UK

**Keywords:** Caregiving, end of life, respiratory, qualitative, thematic synthesis

## Abstract

**Objectives:**

The aim of this review was to identify, review, and synthesize primary qualitative literature to answer the question “what are the roles and experiences of informal caregivers providing care to a person with non-malignant respiratory disease at end of life from the perspectives of the caregiver and recipient of care?”

**Methods:**

This qualitative systematic review was undertaken using thematic synthesis. Electronic databases (British Nursing Database, Cumulative Index to Nursing and Allied Health Literature Plus, Medline, PsycInfo, ProQuest Sociology, Allied and Complementary Medicine Database [AMED]) covering nursing, medicine, and social sciences were systematically searched from inception to October 2024. Studies were included if they reported data on the experiences and roles of caregivers in non-malignant respiratory disease at end of life, from the perspective of the caregiver or the person with non-malignant respiratory disease. Twenty-two papers met the eligibility criteria and were included in the review. Quality assessment was undertaken using the JBI Critical Appraisal Checklist for Qualitative Research.

**Results:**

Thematic synthesis of the data generated five analytical themes: *Caregivers experience shifting identity and new roles; Adaptation is necessary to cope with loss and change; Caregivers need more information and coordinated care services; Emotional effects of caregiving*; and *Future uncertainty and facing death.* The findings illustrated the complexity of the caregiving role and highlight unmet needs during the end of life stage.

**Significance of results:**

This evidence synthesis highlights the significant contribution caregivers make in the lives and deaths of those with non-malignant respiratory disease. Challenges of caregiving in this context increase the stress of caregivers, including unpredictable disease progression and difficult symptoms such as breathlessness. There are persistent inequalities between malignant and non-malignant care pathways. Caregivers would welcome more recognition and information from healthcare professionals to support their role.

## Introduction

The number of people living with chronic conditions, such as non-malignant respiratory disease, is increasing, as is the associated need for informal care (Farquhar 2016). Informal or unpaid care provided by family members and friends is fundamental to the current model of health and social care in the United Kingdom (UK): the contribution by unpaid carers is valued at £162 billion annually (Petrillo and Bennett 2023). The increasing reliance on caregivers, and the economic contribution made by caregivers, makes sustaining this role of great importance to patients, families, and the health and care system. This paper explores the roles and experiences of informal caregivers caring for people with non-malignant respiratory disease at the end of life, to help inform future research and/or service provision.

Non-malignant respiratory diseases are incurable, progressive conditions with significant physical and psychological symptoms and rising global prevalence (Mc Veigh et al. 2019). The definition of non-malignant respiratory disease used in this review will be that of Mc Veigh et al. (2017, 2018, 2019): Interstitial Lung Diseases (ILD), Chronic Obstructive Pulmonary Disease (COPD), and bronchiectasis. These 3 conditions have commonalities in relation to diagnosis in adulthood, symptom burden, and prognosis, therefore suggesting there may be similarities in their impact on caregiver roles and experiences.

There are known inequalities in provision of palliative and end of life care for people with non-malignant respiratory disease (Kim et al. 2019; Butler et al. 2020). Traditionally, palliative care services have provided care for people with cancer diagnoses, and their families. However, most deaths result from causes other than cancer – 71.5% of deaths in 2019 were non-cancer-related – and the need and benefit of palliative care in non-malignant conditions is well recognized (Hospice UK 2021). Improving end of life care for people with non-malignant diagnoses has been highlighted as a priority for service improvement and research (Hospice UK 2021; Hospice 2024; Alliance 2025).

Advanced non-malignant respiratory disease is associated with high biopsychosocial symptom burden including pain, breathlessness, fatigue, and anxiety (Kingston et al. 2020). For family members and friends, high levels of physical disability, along with emotional distress, can result in significant strain (Maddocks et al. 2017; Farquhar 2018). The unpredictable nature of non- malignant respiratory disease progression also hinders accurate prognostication, effective information giving, and timely referral to palliative care services (Pinnock et al. 2011; Boland et al. 2013; Bajwah et al. 2013b; Mc Veigh et al. 2019), meaning less access to potentially beneficial care for people with non-malignant respiratory disease and their caregivers. People with these conditions have been reported to have poorer outcomes at the end of life, compared to those with some malignant conditions. This includes delayed palliative conversations, limited engagement in care planning, higher use of invasive interventions, and increased rates of hospital death (Bloom et al. 2018; Butler et al. 2020; Tavares et al. 2020); all of which can add to caregiver distress and burden (Farquhar 2022). All the above factors may add to caregiver distress and burden (Farquhar 2022). Better understanding of caregivers’ roles in non-malignant respiratory disease during the end of life period (when symptoms and needs intensify) may allow support to be appropriately tailored, thereby benefiting both caregivers and the people they care for.

The aim of this qualitative systematic review was to identify, review and synthesize primary qualitative literature to answer the question “what are the roles and experiences of informal caregivers providing care to a person with non-malignant respiratory disease at end of life from the perspectives of the caregiver and recipient of care?”

## Methods

The protocol for this review was registered on the international systematic review registry (PROSPERO) (registration CRD42023451677), and the completed study is reported in line with Preferred Reporting Items for Systematic Reviews and Meta-Analyses guidelines (Page et al. 2021) where applicable.

The methodology employed in this review was thematic synthesis (Thomas and Harden 2008). This systematic approach is appropriate for identification, review, and synthesis of qualitative studies concerning experiences and perspectives (Thomas and Harden 2008; Barnett-Page and Thomas 2009). Thematic synthesis draws upon methods of primary data analysis (Thomas and Harden 2008) and seeks to identify and translate findings from primary studies and combine them to create themes which offer new information and understanding of the topic.

### Search strategy

A comprehensive search strategy was designed, with speciality librarian input, for the following electronic databases: British Nursing Database, Cumulative Index to Nursing and Allied Health Literature Plus, Medline, PsycInfo, ProQuest Sociology, AMED. Databases were selected to ensure broad coverage of disciplines relevant to the topic (Gusenbauer and Haddaway 2020). Limiters were placed to return only peer-reviewed journals available in English; publication date was limited only by the data parameters of each included database. Searches were run to October 2024 and checked for currency in May 2025.

Full details of search terms are available in Table 1.

**Table 1.** Search terms used in databases searches including Boolean operators

carer* OR caregiver* OR family OR spous*
AND
“Non-malignant respiratory disease” OR “chronic lung disease” OR “chronic respiratory disease” OR COPD OR “chronic obstructive pulmonary disease” OR ILD OR “interstitial lung disease” OR IPF-ILD OR “idiopathic pulmonary fibrosis” OR “diffuse lung disease” OR bronchiectasis
AND
“end of life” OR palliati* OR “supportive care” OR dying OR death OR “terminal care” OR “advanced stage” OR “advanced disease” OR “hospice care”

The SPIDER search strategy tool was used to develop the research question and support retrieval of qualitative evidence (Cooke et al. 2012; (see supplementary materials 1 for more detail). Database searches returned 1644 records. The Rayyan systematic review platform was used for title and abstract screening against pre-defined eligibility criteria (see Table 2) undertaken by KR with discrepancies discussed by the research team. Sixty-six records were included for full text screening. To ensure consistent application of eligibility criteria, the first 20% of full-text records alphabetically by first author were initially screened by both KR and AL and results compared and discussed. Once consistent application of the criteria was assured, the remaining 80% were screened by KR and AL, results compared, and discrepancies resolved. Full inclusion and exclusion criteria are outlined in Table 2.

**Table 2.** Inclusion and exclusion criteria

Inclusion criteria	Exclusion criteria
Studies reporting the roles and experiences of informal caregivers caring for a person with NMRD at the EoL from the perspectives of the caregiver and/or the recipient of care in any setting	Studies where the roles and experiences of caregivers in relation to caregiving are not reported
Studies including patients diagnosed with COPD, ILD or Bronchiectasis and considered to be at the EoL *EoL clearly stated, or disease stage stated as end-stage/severe/advanced disease within the article. If stage not stated, then demographic clinical details which suggest advanced disease e.g., mMRC 3–4/<40% TLCO/COPD GOLD stage 3–4/FVC < 50%	Studies where all recipients of care are not stated to be at the EoL* or data relating to those at EoL cannot be differentiated from a wider sample
Studies where data reported are from participants aged 18 years and over	Studies with a mixed age sample, where the relevant data cannot be differentiated, e.g., *multiple conditions/mixed participant sample*
Peer-reviewed primary research studies using a qualitative or mixed methods design	Studies using solely quantitative research design studies where qualitative data are not reported
Studies available in full text studies available in English	Secondary research/secondary analysis unpublished literature, grey literature, commentary, conference proceedings or abstract only

### Quality assessment

The JBI Critical Appraisal Checklist for Qualitative Research (JBI 2020) was used to assess quality of included studies. KR reviewed all articles; 3 sample articles were reviewed by AL to ensure consistent application of the tool. The aim of quality appraisal was not to determine exclusion or inclusion in the review, rather to enable consideration of quality in relation to findings. The quality assessment is available in Supplementary Materials 2.

### Data extraction

Data were extracted using a standardized form, adapted from Noyes et al. (2018) and used successfully by the authors previously (Rogers et al. 2021). KR extracted data from all articles, with a comparative sample independently extracted and checked by AL (see Table 3).

**Table 3.** Extracted data items

Study details: Author, date/Title/Country/Aims of study
Methods: Study setting/Study design/Recruitment and Sampling strategy/Data collection method/Theoretical framework/approach/background of study/type of analysis/data analysis approach
Study Population: Age ranges/Genders/Socio-economic background/Ethnicity/Education/Occupation/Respiratory condition of cared-for people/Relationships to cared-for people
Ethics: How ethical issues were addressed
Limitations and implications
Findings: All text labelled as “results” or “findings”

### Data synthesis

In thematic synthesis (Thomas and Harden 2008) data synthesis involves a 3-stage process: data are inductively coded line-by-line to identify a unit of meaning and content. The codes are then reviewed for similarities and differences and categorized into “descriptive themes.” Stage 3 requires the researcher (KR) to reconsider the stage 2 descriptive themes in relation to the research question thereby revealing new insights and generating “analytical themes” (Thomas and Harden 2008). Lumivero NVivo qualitative data analysis software was initially used to support stages one and two of the synthesis process (Houghton et al. 2017), the work then continued in MS Word and Excel, allowing flexibility during stage three.

## Results

**Figure 1.**
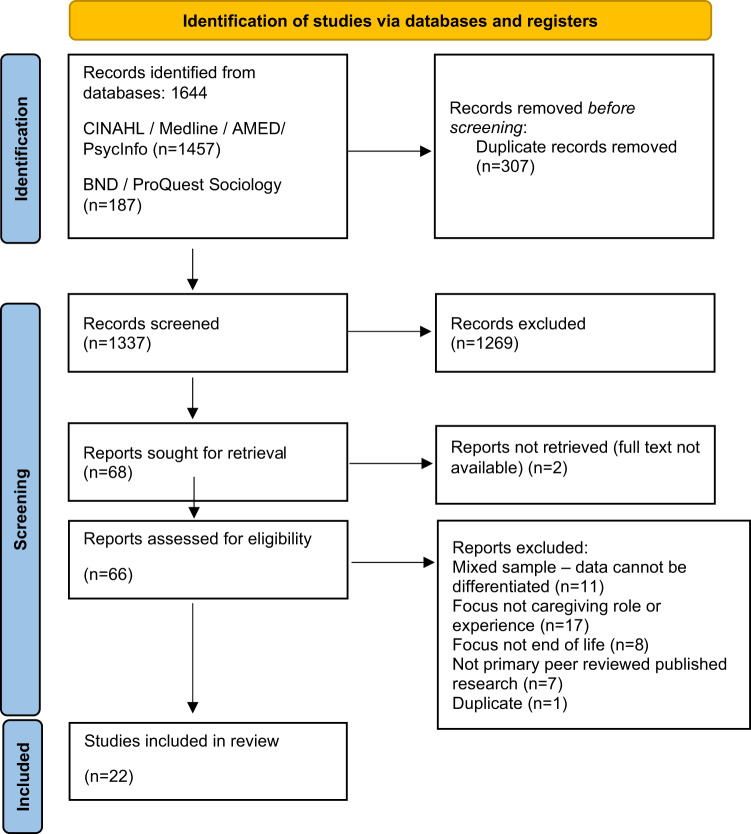
Preferred reporting items for systematic reviews (PRISMA) flow diagram (Page et al. 2021) from searches to May 2025.

Twenty-two articles were identified which met the eligibility criteria and were included for review (see Fig. 1).

### Study characteristics

**Figure 2.**
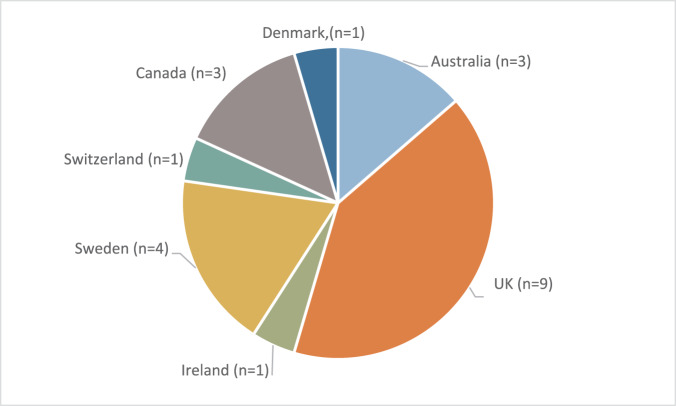
Study location (*n* = the number of studies from each location).

The articles were published between 2001 and 2022 and reported studies from seven countries, with the UK most represented (*n* = 9) (see Fig. 2). Chronic Obstructive Pulmonary Disease was the most represented condition (*n* = 16 studies), 5 studies focused on ILD, and participants affected by bronchiectasis appeared in only 1 study.

A summary table showing characteristics of each included study is given in Table 4.

**Table 4.** Summary table showing characteristics of included studies

Study #	Author/date	Country	Methodology	Research question/aim	Population	Data collection method	Data analysis method
1	Bajwah et al. (2013b)	UK (England)	Qualitative	To explore (1) patients’ and carers’ understanding of their disease (specifically in areas surrounding prognosis), (2) patients’ and carers’ preferences regarding end-of-life planning and (3) patients’, carers’ and health professionals’ views on communication and coordination of care.	8 patients with ILD 4 carers 6 HCPs^1^	Semi-structured interviews	Constant comparative analysis
2	Bajwah et al. (2013b)	UK (England)	Qualitative	To explore the specialist palliative care needs and disease experience of PIF-ILD patients and informal caregivers while enhancing findings with health professionals’ views	8 patients with ILD 4 carers 6 HCPs	Semi-structured interviews	Constant comparative analysis
3	Brown et al. (2012)	Australia	Qualitative	To explore needs for services and support for people with advanced COPD and discuss advance care planning	15 patients with advanced COPD and their 8 carers	2 semi-structured interviews conducted 6 months apart	Thematic analysis
4	Cawley et al. (2014)	UK (Scotland)	Qualitative	To identify events that potentially could act as triggers for provision of supportive and palliative care in COPD	21 patients with COPD 13 informal carers 18 HCPs	In-depth participant-led interviews at 4 timepoints over 18 months	Thematic and content analysis
5	Egerod et al. (2019)	Denmark	Qualitative, explorative, descriptive design	To describe the experience of f-ILD patients’ spouses during the final stages of illness and up to the first year after the patient’s death to investigate if palliative care could ease the transition and prevent Prolonged Grief Disorder.	20 bereaved spouses of people with ILD	Semi-structured in-depth interviews, field notes, diaries, Grief questionnaire PG-13^2^	Thematic analysis
6	Ek et al. (2015)	Sweden	Qualitative descriptive design	To retrospectively describe the final year of life for patients with advanced COPD with a focus on dying and death from the perspective of relatives.	13 bereaved relatives of people with COPD	Interviews and descriptions of the course of the patients’ disease.	Qualitative content analysis
7	Ek et al. (2011)	Sweden	Hermeneutic phenomenology	To illuminate couples’ experiences of living together when one partner has advanced COPD treated by means of long-term oxygen therapy.	4 couples including a person with COPD	Interviews at 4 time points	Dialectal analysis
8	Elkington et al. (2004)	UK (England)	Qualitative	To explore the experience of the last year of life of COPD from the perspective of bereaved relatives	28 bereaved caregivers of people with COPD	In-depth interviews	Framework analysis
9	Ferreira et al. (2020)	Australia	Qualitative	To understand the experience of living with, and responding to, severe chronic breathlessness in people with COPD from the perspective of the patient and their carer.	9 patients with COPD and their carers	Semi-structured interviews	Grounded theory using constant comparative approach
10	Fusi-Schmidhauser et al. (2020)	Switzerland	Qualitative	To explore the experiences of patients and informal carers in living with advanced COPD and to understand their awareness about palliative care pro-vision in advanced COPD.	20 patients with COPD 20 caregivers	Semi-structured interviews	Thematic analysis
11	Guthrie et al. (2001)	UK (England)	Qualitative	To gain an impression of the experience and behavior, feelings and judgements, of patients whose daily lives are affected by severe COPD.	20 patients with COPD	In-depth interviews at 3 times points	5 stage analysis described
12	Hasson et al. (2008)	UK (Northern Ireland)	Qualitative descriptive	To explore the potential for palliative care among people living with advanced chronic obstructive pulmonary disease	13 patients with COPD	In-depth interviews	Content analysis
13	Hasson et al. (2009)	UK (Northern Ireland)	Qualitative descriptive	To explore the palliative and end of life care needs and experiences of bereaved family members who cared for a person with advanced COPD in the home.	9 bereaved carers of people with COPD	Semi-structured interviews	Content analysis
14	Kalluri et al. (2022)	Canada	Qualitative	To explore perspectives of IPF patients, family caregivers, and healthcare professionals on ACP-related experiences to understand and inform an ACP framework.	5 patients with IPF 5 caregivers 10 HCPs	Semi-structured interviews	Inductive analysis
15	Mc Veigh et al. (2017)	Ireland and UK (Northern Ireland)	Qualitative	To explore specialist and generalist palliative care provision for people with non-malignant respiratory disease, in rural and urban areas in the North and Republic of Ireland	17 bereaved carers of people with NMRD 18 HCPs	Semi-structured interviews (carers) Focus groups (HCPs)	Thematic analysis
16	Molzahn et al. (2021)	Canada	Narrative enquiry – social constructionist perspective	How do people diagnosed with advanced COPD describe living with impending death? How do family members describe the experience of caring for a person diagnosed with advanced COPD and living with impending death?	16 patients with COPD 7 relatives	3 in-depth interviews	Thematic analysis
17	Philip et al. (2014)	Australia	Qualitative	To explore the experiences of current and bereaved informal carers of patients with severe COPD with a particular focus upon their information needs and views of palliative care.	9 current carers and 10 bereaved carers of people with COPD	In-depth semi-structured interviews	Thematic analysis
18	Pooler et al. (2018)	Canada	Qualitative narrative approach	To explore bereaved caregivers’ experiences and perceptions of an early integrated palliative approach implemented at a Multidisciplinary Interstitial Lung Disease Clinic	8 bereaved caregivers of people with ILD	Open-ended interviews	Thematic and content analysis
19	Seamark et al. (2004)	UK (England)	Phenomenology	To explore the experiences of patients living with severe COPD and the impact on their carers.	10 patients with COPD and their carers	Semi-structured interviews	Interpretative phenomenological analysis
20	Spence et al. (2008)	UK (Northern Ireland)	Qualitative descriptive	To explore the specific care needs of informal caregivers of patients with advanced COPD	7 caregivers of people with COPD	Semi-structured interviews	Content analysis
21	Strang et al. (2019)	Sweden	Qualitative	To describe perceptions of caregiving support from healthcare to informal caregivers of patients with COPD from two perspectives: (1) the family caregiver’s perspective and (2) the perspective of healthcare staff.	36 Caregivers of people with COPD 17 HCPs	Focus groups and semi-structured interviews	Content analysis
22	Strang et al. (2018)	Sweden	Qualitative	To explore what impact severe COPD has on family members’ everyday lives with regard to caregiving fora patient in advanced disease, and how the relationship between the patient and the family caregiver is affected.	35 Caregivers of people with COPD	Focus groups and semi-structured interviews	Content analysis

Notes:

1 Where HCPs form part of the sample, data collected from HCPs was not extracted for synthesis.

2 Mixed method data collection, only qualitative data was extracted for synthesis.

Table designed using ENTREQ framework for reporting the synthesis of qualitative health research Tong *et al*. (2012). [year of publication, country, population, number of participants, data collection, methodology, analysis, research questions]

### Data synthesis

The data synthesis process generated 5 analytical themes:

1. Caregivers experience a shift in identity and new roles
2. Adaptation is necessary to cope with loss and change
3. Caregivers need more information and coordinated care services
4. Emotional effects of caregiving
5. Future uncertainty and facing death

See Table 5 for individual study contributions to the analytical themes.

**Table 5.** Individual study contributions to the analytical themes

Studies	Caregivers experience shifting identity, and new, additional roles	Adaptation is necessary to cope with loss and change	Caregivers need more information and coordinated care services	Emotional effects of caregiving	Future uncertainty and facing death
Bajwah et al. (2013a)	✓	✓	✓		✓
Bajwah et al. (2013a)	✓	✓	✓	✓	
Brown et al. (2012)	✓		✓		✓
Cawley et al. (2014)	✓		✓	✓	
Egerod et al. (2019)		✓	✓	✓	✓
Ek et al. (2011)	✓	✓	✓	✓	✓
Ek et al. (2015)	✓	✓	✓	✓	✓
Elkington et al. (2004)	✓	✓		✓	✓
Ferreira et al. (2020)	✓	✓		✓	✓
Fusi-Schmidhauser et al. (2020)	✓	✓	✓	✓	✓
Guthrie et al. (2001)	✓	✓			
Hasson et al. (2008)	✓	✓	✓	✓	✓
Hasson et al. (2009)	✓	✓	✓	✓	✓
Kalluri et al. (2022)	✓		✓		✓
Mc Veigh et al. (2017)	✓	✓	✓		✓
Molzahn et al. (2021)		✓		✓	✓
Philip et al. (2014)	✓	✓	✓	✓	✓
Pooler et al. (2018)	✓	✓	✓	✓	✓
Seamark et al. (2004)	✓	✓		✓	
Spence et al. (2008)	✓	✓	✓	✓	✓
Strang et al. (2018)	✓	✓	✓	✓	✓
Strang et al. (2019)	✓	✓	✓	✓	

### Caregivers experience a shift in identity and new roles

Caregiving caused a gradual change in the nature of the relationship between the cared-for person and the caregiver; participants reported emotional and functional changes to relationships: *“It feels like I’ve lost my friend. She is not the same person anymore. She has become more selfish and never asks me how I feel, but all focus should be on her”* (Caregiver quote; Strang et al. 2018). These changes related to the shift in responsibilities as the cared-for person became more unwell and required more support: *“I want a relationship with my husband. I don’t want to check his medications, or check clothes, no, I just want to have a good time with him”* (Caregiver quote; Strang et al. 2019).

Caregivers described taking on a new identity, although this identity was not always initially acknowledged or accepted positively: *“Carers described a lack of choice, even for some, a lack of awareness, until they suddenly realized they were fulfilling a significant caring role”* (Author interpretation; Philip et al. 2014). *“Informal caregivers like Penny, wife of James, struggled to adapt to the increasing reliance of her husband on her time and emotional well-being. She felt suffocated and frustrated by his neediness” (*Author interpretation; Bajwah et al. 2013a). Their responsibilities were not only related to the care needs of the cared-for person, but also to the domestic roles previously held by the cared-for person *“I’m doing a thousand jobs as well. I’m just going crazy because you don’t get to the end of it… We’re nurses, we’re doctors, we’re housewives, we’re cooks, we’re gardeners. We’re shopping”* (Caregiver quote; Seamark et al. 2004).

Caregivers fulfilled a range of roles and in some cases had developed expert knowledge of the condition and associated treatments. Alongside practical, everyday help such as care for personal hygiene, errands, and household duties like shopping and cleaning, caregivers assumed more complex responsibilities. Caregivers became information-givers and decision-makers in the care of the person: *“I said she’s not improving much, doctor … She had the bipap here before when she was about this stage and the nurse said, Yeah that might be a good idea, and the doctor put the bipap on.”* (Caregiver quote; Philip et al. 2014). Caregivers were relied upon to relay vital health details to healthcare professionals and in some cases to seek out information about the disease; this proved upsetting when relating to disease severity and prognosis: *“Myself and my husband got on the internet and found out ‘well actually life spans 5 years,’ she had no idea, no one’s even told her that (…) so we go ‘how do we tell her this’ (…) so actually the actual breaking the news was myself…”* (Caregiver quote; Bajwah et al. 2013b).

Caregivers were required to make complicated judgements about when to seek support of healthcare services and often held responsibility for implementing decisions about future care, when advance care planning had taken place: *“I think this one is about when they don’t want to be revived, if you got so bad … it is for him to give permission for me to make his decisions on his behalf knowing what he wants?”* (Caregiver quote; Brown et al. 2012). This role was reported both negatively as a stressor but also positively as a comfort, knowing they could facilitate the person’s wishes: *“Because when he was in the hospital, he was in the hospital for a week and he said to me, ‘Don’t ever, ever send me to the hospital to pass away.’ And I said ‘I won’t.’ And I didn’t and I’m glad*…” (Caregiver quote; Pooler et al. 2018).

### Adaptation is necessary to cope with loss and change

Caregivers felt a responsibility to maintain everyday life and to do this, adaptation was needed to accommodate the effects of the illness, in particular breathlessness. Adaptations were made in many aspects of life in relation to daily activities and how the environment was arranged: *“…it’d be easier if she stayed in this room so we made that into a bed. And what we did, I mean, the toilet is literally right there, kitchen is out there”* (Caregiver quote; Elkington et al. 2004). The nature of relationships, and the expectations caregivers had of their futures were disrupted by the illness and accompanied by a sense of loss, for example, a participant with COPD commented, *“Maybe the hardest thing is that I’m just not up to having sex anymore [pauses for breath], I’m too sick and that’s really hard [cries]”* (Ek et al. 2011). *The participant’s partner acknowledged the need to adapt: “You just have to accept things as they are. In the long run it’s not 100 percent important either. Now we are thankful as long as we still have each other”* (Ek et al. 2011). Adaptation was ongoing and dynamic in relation to deterioration in the health of the cared-for person and increasing limits on their functional ability: *“We miss travelling. We loved to go places and every so often, I forget and I said ‘why aren’t we going down to the warmer climate?’ Now that we have a few pennies … and then we realize … the insurance doesn’t cover his illness”* (Caregiver quote; Molzahn et al. 2021).

To cope with increased demands of caregiving, various coping strategies were employed. Peer support from other caregivers was viewed positively by those who engaged with it and was reported to help with both practical and emotional aspects of caregiving. Maintaining hope and a positive attitude also contributed to coping with caregiving: *“You can’t get stuck over the impossible, instead you must focus on things that are possible to do. Otherwise it would be just miserable”* (Caregiver quote; Strang et al. 2018). Although in relation to deteriorating health of the cared-for person, this hope was often short-lived: *“I don’t think I realized how sick my husband was toward the end, and I remained optimistic although we could see that it was unlikely he would be able to get up again”* (Caregiver quote; Egerod et al. 2019).

The constant nature of caregiving was apparent, and caregivers reported impact on their own health and social life in the course of caregiving: *“I collapse. I take sedatives, I sleep poorly, my body aches.. this burden to always take care, I can’t do it anymore. So I have sought help for myself, because I can’t take it anymore, not physically not mentally, I feel like I’m collapsing”* (Caregiver quote; Strang et al. 2019). *“We always had a lot of friends and family in our home … this is the past … he doesn’t want anyone at home … it is so sad and I feel lonely* …” (Caregiver quote; Fusi-Schmidhauser et al. 2020). The cared-for people recognized the burden of caregiving on their family member and worried for the health of that person: “‘*I think that the one that suffers most is (my wife) because she’s been hard at it for eighteen months looking after me.’ ‘She’s a sticker, she treats me well and she looks after me, but it’s taken a toll, it’s taken a toll’*” (Person with COPD; Seamark et al. 2004).

### Caregivers need more information and co-ordinated care services

Caregivers felt they needed more information than was provided by healthcare professionals, particularly in relation to the progression of the disease, the treatments being used (e.g., oxygen therapy), and conversations about the end of life: *“There was no information and it was really hard to find and it felt like we were completely on our own … From what I found online, it was scary”* (Caregiver quote; Kalluri et al. 2022).

Information was important to support the adaptations needed in response to changes in the health of the person with non-malignant respiratory disease. Being able to talk with healthcare professionals was valuable for caregivers’ own needs, as well as the needs of the cared-for person. Information about available support services and practical issues, such as financial advice, was viewed as lacking and caregivers had to seek sources of information beyond healthcare professionals: *“What would have been helpful and comforting for me [is] to have been fully informed. [The patient] didn’t always want to think about it, but I needed to so I could work out what to do”* (Caregiver quote; Philip et al. 2014). Contact with healthcare professionals was appreciated and acted as a support in itself, but did not always happen, or did not happen in a timely way according to caregivers.

Caregivers tended not to emphasize their caregiving role and much of the role remained invisible to those outside the home. This lack of recognition of the role left some caregivers without suitable support: *“No, no one has asked about me. Sometimes I wish someone would ask how we’re doing here at home*” (Caregiver quote. Ek et al. 2011).

Palliative care services were not familiar to all participants, but the concept of holistic care was seen as relevant to care of people with non-malignant respiratory disease from early in the disease trajectory. Issues arose with, and between, primary and secondary care services: secondary care such as respiratory clinics were valued for their specialist skills but could be far from home. Primary and community services did not always provide continuity, and this impacted upon where people were cared for and where they died: *“She did die in hospital, she did indeed and that’s part of my hurt, calling the ambulance and letting her die in there. She would have loved to have died at home. I think that if we had had more support at home she could have died at home*” (Caregiver quote; Mc Veigh et al. 2017).

Some participants were able to access local healthcare professionals when needed but others reported not knowing who to contact for support: *“Even knowing who to call, or someone to call, would be really nice because when you’re the caregiver it’s not a 9 to 5. It’s 24 hours”* (Caregiver quote; Pooler et al. 2018).

### Emotional effects of caregiving

Caregiving impacted upon the emotional state of caregivers, in the main this was a negative impact although some positives were identified. Watching the person with non-malignant respiratory disease struggle with symptoms, particularly breathlessness, was very difficult for caregivers who found it frightening: *“Yes, it is a horror seeing your relative trying to breathe but not getting any air. You cannot do anything! You just try to calm her down and wait for the ambulance”* (Caregiver quote. Strang et al. 2018).

As the person’s condition deteriorated, caregivers reported increased anxiety and doubt about their ability to provide adequate care: *“If she gets breathless, I am getting anxious and distressed…I really ask myself if I am the right person to look after her*…” (Caregiver quote; Fusi-Schmidhauser et al. 2020). There were feelings of blame and guilt between caregivers and cared-for people, for some caregivers the smoking-related etiology of COPD caused resentment toward the cared-for person, blaming them for their illness: *“… but if he would stop smoking, he may be able to keep 40% of his lung function … but nothing, he keeps smoking …”* (Caregiver quote; Fusi-Schmidhauser et al. 2020). Both caregivers and cared-for people felt guilt about their situation, cared-for people felt guilty for causing burden, and caregivers worried about not doing enough or not making the right decisions around care and treatment: *“I haven’t have trouble accepting that he’s gone, but I just can’t accept the way he died … why didn’t I go and get the nurses? It was my biggest mistake”* (Caregiver quote. Egerod et al. 2019).

Despite the evident emotional toll of caregiving, some caregivers expressed feelings of pride from fulfilling a duty to care for a family member, or advocating for the person to have their wishes met at end of life: *“I just love him and I find that every day when I see him, what else could I do to try and make him a wee bit … better? It’s very satisfying to know that he appreciates what I do and it’s nice to know that you are helping someone”* (Caregiver quote; Spence et al. 2008).

### Future uncertainty and facing death

Although many participants understood the terminal nature of non-malignant respiratory disease, the speed with which their family member deteriorated and died was shocking. The unpredictability of the disease progression made facing death difficult: *“She could be fit in the morning, and then they would phone in the evening to say that I had better come because she hasn’t got long to live, and it continued like that for a very long time*” (Caregiver quote; Ek et al. 2015).

As caregivers became accustomed to their family member surviving recurrent exacerbations of their condition, the final deterioration was not always identified as the end of their life: *“We [wife and patient] had no idea that he might die. It just didn’t occur to us. Instead, we thought he would become fit as usual and return home, because that is what had happened on previous occasions”* (Caregiver quote; Ek et al. 2011).

Place of death was important for caregivers reflecting upon the death. Ensuring they died in their preferred place was seen as honoring that person’s wishes: “*But I’m glad I did what I did (pause) because he didn’t want to pass away at the hospital. [emphatic] He wanted to pass away at home. (long pause) … he said to me, ‘Don’t ever, ever send me to the hospital to pass away.’ And I said ‘I won’t.’ And I didn’t and I’m glad”* (Caregiver quote; Pooler et al. 2018).

Both participants and caregivers feared the symptoms of dying, particularly severe breathlessness, and caregivers sought to reassure their family member that they would alleviate these symptoms when the time came: *“My greatest worry was that I couldn’t keep my promise to him that he wouldn’t suffocate … he took his last breath at three in the morning, death came peacefully and it comforted us”* (Caregiver quote; Egerod et al. 2019). Caregivers saw future care planning and decisions about what to do in an emergency as helpful, but these were not always in place due to difficulty in discussing sensitive subjects: *“… You’ve probably got to talk about it. What if a decision had to be made? At the moment he’s very lucky, but if we had an emergency and it’s life threatening, well that would have to be my decision. I’m not sure what I would say. I don’t know. We haven’t talked about it”* (Caregiver quote; Philip et al. 2014).

## Discussion

This review aimed to identify, collate, and synthesize the qualitative evidence in relation to the roles and experiences of informal caregivers when caring for someone with non-malignant respiratory disease at the end of life. To the best of our knowledge, it is the first review to focus solely on caregivers in this particular context. Respiratory conditions have high global mortality rates, yet this patient group experience a lack of access to specialist palliative care when compared to people with cancer (Janssen et al. 2023). Focus on this group of caregivers is highly relevant as demand for informal care remains high and the contribution of caregivers is essential to current healthcare systems. This review found that the experience of caregiving in non-malignant respiratory disease at the end of life was characterized by change and loss as caregivers face an uncertain future; more information and support are needed to sustain their caregiving role.

The experience of shifting identity and roles, and coping with loss and change, are not experiences unique to caregiving in non-malignant respiratory disease. For example, Catchpole and Garip (2021) found role and identity changes in people caring for a person with chronic fatigue syndrome (CFS). As in the current review, caregivers in CFS also reported loss of previous life, and frustration in their caregiving role (Catchpole and Garip 2021). The current review found participants described losing aspects of their previous relationship because of the illness and caregiving role. Similarly, Broady (2015) discussed how caring for a spouse with declining health creates imbalance in the spousal relationship as one partner becomes increasingly dependent, and Zhu et al. (2023) reported that family caregivers in advanced cancer found additional roles to be a characteristic of caregiving, spanning both practical and emotional aspects.

When compared with the findings of this review, the conclusions of Zhu et al. (2023) demonstrate commonality of experience between caregivers for people with cancer and for people with non-malignant respiratory disease: for example, adaptation and coping strategies across the groups were similar such as, adopting a positive attitude and seeking social support. Also echoing the findings of the current review, Zhu et al. (2023) noted that although social support was viewed positively, family caregivers reported feeling isolated with reduced social contacts outside the home.

These similarities between caregiving in cancer, and caregiving in non-malignant respiratory disease, demonstrate a widespread need to improve caregiver support and suggest that characteristics of the caregiving experience at the end of life may transcend clinical diagnosis. Despite this suggestion, it should be noted that people with advanced cancer are more likely to receive specialist palliative care input (Butler et al. 2020) which may include support for caregivers; further highlighting the long-standing inequality and disadvantage of people with, and caring for those with, a non-malignant diagnosis (Care Quality Commission 2016; National Confidential Enquiry into Patient Outcome and Death 2024).

The current review clearly demonstrates that caregivers have unmet needs. This finding resonates with the work of Farquhar (2016, 2018, 2022) which illustrates the importance of understanding and assessing the needs of caregivers. In particular, the current review highlights unmet needs in relation to information and access to care services. Caregivers report lacking information about practical aspects such as finances, and medical issues such as disease progression and end of life planning. Häger Tibell et al. (2024) found that support and information sharing by healthcare professionals led to higher levels of caregiver preparedness for caregiving and for death in advanced cancer, demonstrating the value of contact and communication between families and healthcare professionals. Mirroring findings in the current review, a scoping review of caregiver needs in pulmonary fibrosis also highlighted the importance of information about disease progression, alongside practical advice and the desire to discuss future care planning (Klein et al. 2021).

Caregiving in advanced non-malignant respiratory disease can be highly emotive. Feelings of fear, guilt and responsibility are evident for caregivers who are present alongside people as their health deteriorates, and symptoms worsen. Living with COPD can illicit feelings of guilt on the part of the ill person and blame from the caregiver for a perceived “self-inflicted” illness; this may lead to people with COPD feeling unworthy of support (Jerpseth et al. 2021), further complicating the relationship between caregiver and cared-for. In a narrative review of caregiving at end of life, Nicholls et al. (2025), found feelings of caregiver reward and satisfaction similar to those reported by some participants in the current review. Such feelings often related to successfully upholding the dying persons’ wishes about preferred place of death, highlighting the need for conversations to establish preferences for future care. The current review illustrates the emotional and practical complexity of caregiving in non-malignant respiratory disease, particularly in the context of known inequalities between malignant and non-malignant care provision which may impact the experience of caregivers.

Taylor (2017) suggests that prognostic conversations commonly precede advance care planning meaning the unpredictability of disease progression and challenge of prognostication in non-malignant respiratory disease may impede timely referrals to palliative care services and cause uncertainty and distress for caregivers (Kim et al. 2019; Ng et al. 2024). In the current review, caregivers were often aware of the incurable, progressive nature of the condition but advance care planning, whether with healthcare professionals or in conversation with the ill person, did not always take place. When advance care planning had taken place, caregivers felt satisfied that they could fulfil the wishes of the ill person. It has been further asserted that advance care planning may improve bereavement adjustment for caregivers, as it involves emotional preparation for the death and provides clarity for end of life decision-making (Falzarano et al. 2021). In exploring how responsibility shapes the role of family caregivers in Motor Neurone Disease, Wilson et al. (2025) also highlight bereavement risks associated with caregiving. As in the current review, complex roles, such as decision-maker, were found to be undertaken by caregivers and Wilson et al. (2025) suggest that the loss of these roles, in addition to their relative’s death, could exacerbate the bereavement experience. Given the potential significant health risks of bereavement, such as physical health problems, poor psychological health and excess mortality, as highlighted by Stroebe et al. (2007), attention on bereavement adjustment and promotion of wellbeing for caregivers is an important consideration for service providers.

### Limitations and strengths

Whilst systematic searches were conducted, including follow-up searches to locate newly published work, a possible limitation of this review is that relevant studies may not have been identified, for example those reported in unpublished literature. However, sufficient studies were included in the review to address the research question. Furthermore, none of the included studies was identified as being of especially low quality, although it is noted that the philosophical standpoint, beyond a statement of qualitative methodology, was noted in only 4 of the studies (Seamark et al. 2004; Ek et al. 2011; Mc Veigh et al. 2017; Molzahn et al. 2021). Nonetheless, methods were congruent with methodology, and appropriate for the research question or study aims, in all cases.

The authors acknowledge that COPD was the most represented condition in the data included in the current review. ILD and particularly bronchiectasis were less well represented. Whilst COPD is a more prevalent condition (World Health Organization 2024), the experiences of those with ILD and bronchiectasis deserve attention, and more research is warranted in these conditions in future.

It could be argued that including international studies in the current review may limit the transferability of findings across UK health and care systems; however, all studies were from countries with similarly developed healthcare systems, and themes were crosscutting across all the regions represented by the studies.

Thematic synthesis relies upon the researcher to synthesize and interpret data as presented by the primary authors to generate new insights. The subjective value of qualitative interpretation is acknowledged, and the research process has been reported transparently to demonstrate rigor. The lead author is a registered nurse with experience in the care of people dying with chronic, non-malignant conditions and their families. This experience undoubtedly influenced the research process and was reflected upon individually, and within the research team during the study, to examine the work and interrogate potential underlying assumptions. This reflexive practice is a particular strength of the current review, as was the input of public contributors as part of the research team who endorsed the overall topic, contributed to decisions on terminology, and reviewed findings during the synthesis stage.

### Conclusion

The current review sheds light on the multifaceted roles and deeply emotional experiences of informal caregivers providing care to a person with non-malignant respiratory disease at end of life, illustrating the highly unpredictable and complex nature of caregiving in non-malignant respiratory disease. Unmet informational needs appear to be a pervasive part of caregiver experience highlighting the need for awareness and recognition of caregivers by those working within the health and social care systems. The significant contribution of caregivers, at an individual and population level, against a backdrop of continuing inequality, clearly illustrates the need for strengthened efforts to improve support for caregivers and in turn the people they care for.

## Supporting information

10.1017/S1478951526101643.sm001 Rogers et al. supplementary material 1 Rogers et al. supplementary material

10.1017/S1478951526101643.sm002 Rogers et al. supplementary material 2 Rogers et al. supplementary material

## Supplementary material

The supplementary material for this article can be found at https://doi.org/10.1017/S1478951526101643.

## References

[ref1] Alliance JL (2025) *Research priorities for palliative and end of life care*. Available at: https://www.jla.nihr.ac.uk/priority-setting-partnerships/palliative-and-end-of-life-care-refresh#tab-65236 (accessed 21 March 2025).

[ref2] Bajwah S, Higginson I, Ross J, et al. (2013a) The palliative care needs for fibrotic interstitial lung disease: A qualitative study of patients, informal caregivers and health professionals. *Palliative Medicine* 27(9), 869–876. doi: 10.1177/0269216313497226 23885010

[ref3] Bajwah S, Koffman J, Higginson I, et al. (2013b) I wish I knew more …’ the end-of- life planning and information needs for end-stage fibrotic interstitial lung disease: Views of patients, carers, and health professionals. *BMJ Supportive and Palliative Care* 3, 84–90. doi: 10.1136/bmjspcare-2012-000263 24644332

[ref4] Barnett-Page E and Thomas J (2009) Methods for the synthesis of qualitative research: A critical review. *BMC Medical Research Methodology* 9(1), 59–59. doi: 10.1186/1471-2288-9-59 19671152 PMC3224695

[ref5] Bloom C, Slaich B, Morales D, et al. (2018) Low uptake of palliative care for COPD patients within primary care in the UK. *European Respiratory Journal* 51(2), 1701879. doi: 10.1183/13993003.01879-2017 29444916 PMC5898942

[ref6] Boland J, Martin J, Wells A, et al. (2013) Palliative care for people with non-malignant lung disease: Summary of current evidence and future direction. *Palliative Medicine* 27(9), 811–816. doi: 10.1177/0269216313493467 23838376

[ref7] Broady TR (2015) The carer persona: Masking individual identities. *Persona Studies* 1(1), 65–75. doi: 10.21153/ps2015vol1no1art392

[ref8] Brown M, Brooksbank M, Burgess T, et al. (2012) The experience of patients with advanced chronic obstructive pulmonary disease and advance care-planning: A South Australian perspective. *Journal of Law and Medicine* 20(2), 400–409. 23431856

[ref9] Butler S, Ellerton L, Gershon A, et al. (2020) Comparison of end-of-life care in people with chronic obstructive pulmonary disease or lung cancer: A systematic review. *Palliative Medicine* 34(8), 1030–1043. doi: 10.1177/0269216320929556 32484762

[ref10] Care Quality Commission (2016) A different ending. Addressing inequalities in end of life care. Available at: https://www.cqc.org.uk/sites/default/files/20160505%20CQC_EOLC_OVERVIEW_FINAL_3.pdf (accessed 21 March 2025).

[ref11] Catchpole S and Garip G (2021) Acceptance and identity change: An interpretative phenomenological analysis of carers’ experiences in myalgic encephalopathy/chronic fatigue syndrome. *Journal of Health Psychology* 26(5), 672–687. doi: 10.1177/1359105319834678 30895822

[ref12] Cawley D, Billings J, Oliver D, et al. (2014) Potential triggers for the holistic assessment of people with severe chronic obstructive pulmonary disease: Analysis of multiperspective, serial qualitative interviews. *BMJ Supportive & Palliative Care* 4(2), 152–160. doi: 10.1136/bmjspcare-2013-000629 24681560

[ref13] Cooke A, Smith D and Booth A (2012) Beyond PICO: The SPIDER tool for qualitative evidence synthesis. *Qualitative Health Research* 22(10), 1435–1443. doi: 10.1177/1049732312452938 22829486

[ref14] Egerod I, Kaldan G, Shaker S, et al. (2019) Spousal bereavement after fibrotic interstitial lung disease: A qualitative study. *Respiratory Medicine* 146, 129–136. doi: 10.1016/j.rmed.2018.12.008 30665511

[ref15] Ek K, Andershed B, Sahlberg-Blom E, et al. (2015) “The unpredictable death”—The last year of life for patients with advanced COPD: Relatives’ stories. *Palliative and Supportive Care* 13(5), 1213–1222. doi: 10.1017/S1478951514001151 25315360

[ref16] Ek K, Ternestedt B, Andershed B, et al. (2011) Shifting life rhythms: Couples’ stories about living together when one spouse has advanced chronic obstructive pulmonary disease. *Journal of Palliative Care* 27(3), 189–197. doi: 10.1177/082585971102700302 21957795

[ref17] Elkington H, White P, Addington-Hall J, et al. (2004) The last year of life of COPD: A qualitative study of symptoms and services. *Respiratory Medicine* 98(5), 439–445. doi: 10.1016/j.rmed.2003.11.006 15139573

[ref18] Falzarano F, Prigerson HG and Maciejewski PK (2021) The role of advance care planning in cancer patient and caregiver grief resolution: Helpful or harmful? *Cancers* 13(8), 1977. doi: 10.3390/cancers13081977 33924214 PMC8074595

[ref19] Farquhar M (2016) Supporting Informal Carers. In Bausewein C, Currow D and Johnson M (eds), *Palliative Care in Respiratory Disease*. Sheffield: European Respiratory Society, pp. 51–69.

[ref20] Farquhar M (2018) Assessing carer needs in chronic obstructive pulmonary disease. *Chronic Respiratory Disease* 15(1), 26–35. doi: 10.1177/1479972317719086 28685601 PMC5802659

[ref21] Farquhar M (2022) Improving support of informal carers of respiratory patients. *Respirology* 27(2), 103–104. doi: 10.1111/resp.14175 34708489

[ref22] Ferreira D, Kochovska S, Honson A, et al. (2020) Two faces of the same coin: A qualitative study of patients’ and carers’ coexistence with chronic breathlessness associated with chronic obstructive pulmonary disease (COPD). *BMC Palliative Care* 19(1), 64–64. doi: 10.1186/s12904-020-00572-7 32375747 PMC7203967

[ref23] Fusi-Schmidhauser T, Froggatt K and Preston N (2020) Living with advanced chronic obstructive pulmonary disease: A qualitative interview study with patients and informal carers. *Journal of Chronic Obstructive Pulmonary Disease* 17(4), 410–418. doi: 10.1080/15412555.2020.1782867 32586144

[ref24] Gusenbauer M and Haddaway N (2020) Which academic search systems are suitable for systematic reviews or meta‐analyses? evaluating retrieval qualities of google scholar, pubmed, and 26 other resources. *Research Synthesis Methods* 11(2), 181–217. doi: 10.1002/jrsm.1378 31614060 PMC7079055

[ref25] Guthrie S, Hill K and Muers M (2001) Living with severe COPD. A qualitative exploration of the experience of patients in Leeds. *Respiratory Medicine* 95(3), 196–204. doi: 10.1053/rmed.2000.1021 11266237

[ref26] Häger Tibell L, Årestedt K, Holm M, et al. (2024) Preparedness for caregiving and preparedness for death: Associations and modifiable thereafter factors among family caregivers of patients with advanced cancer in specialized home care. *Death Studies* 48(4), 407–416. doi: 10.1080/07481187.2023.2231388 37441803

[ref27] Hasson F, Spence A, Waldron M, et al. (2008) I cannot get a breath: Experiences of living with advanced chronic obstructive pulmonary disease. *International Journal of Palliative Nursing* 14(11), 526–531. doi: 10.12968/ijpn.2008.14.11.31756 19060802

[ref28] Hasson F, Spence A, Waldron M, et al. (2009) Experiences and needs of bereaved carers during palliative and end-of-life care for people with chronic obstructive pulmonary disease. *Journal of Palliative Care* 25(3), 157–163. doi: 10.1177/082585970902500302 19824276

[ref29] Hospice UK (2021) Equality in hospice and end of life care: Challenges and change. Available at: https://hospiceuk-files-prod.s3.eu-west-2.amazonaws.com/s3fs-public/2021-10/Equality%20in%20hospice%20and%20end%20of%20life%20care%20-%20May%202021_0.pdf (accessed 18 November 2024).

[ref30] Hospice UK (2024) Hospice care for all, for now and forever. Available at: https://hospiceuk-files-prod.s3.eu-west-2.amazonaws.com/s3fs-public/2024-04/Hospice%20Strategy%20Document%2022April2024_0.pdf (accessed 18 November 2024).

[ref31] Houghton C, Murphy K, Meehan B, et al. (2017) From screening to synthesis: Using nvivo to enhance transparency in qualitative evidence synthesis. *Journal of Clinical Nursing* 26(5–6), 873–881. doi: 10.1111/jocn.13443 27324875

[ref32] Janssen D Bajwah S, Boon M, et al. (2023) European Respiratory Society clinical practice guideline: palliative care for people with COPD or interstitial lung disease. *European Respiratory Journal* 62(2), 2202014. doi: 10.1183/13993003.02014-2022. 37290789

[ref33] JBI (2020) *JBI Critical Appraisal Checklist for Qualitative Research*. Available at: https://jbi.global/critical-appraisal-tools. (accessed 18 November 2024).

[ref34] Jerpseth H, Knutsen I, Jensen K, et al. (2021) Mirror of shame: Patients experiences of late‐stage COPD. A qualitative study. *Journal of Clinical Nursing* 30(19–20), 2854–2862. doi: 10.1111/jocn.15792 33934413

[ref35] Kalluri M, Orenstein S, Archibald N, et al. (2022) Advance care planning needs in idiopathic pulmonary fibrosis: A qualitative study. *American Journal of Hospice & Palliative Medicine* 39(6), 641–651. doi: 10.1177/10499091211041724 34433294 PMC9082969

[ref36] Kim JW, Atkins C and Wilson AM (2019) Barriers to specialist palliative care in interstitial lung disease: A systematic review. *BMJ Supportive and Palliative Care* 9(2), 130–138. doi: 10.1136/bmjspcare-2018-001575 30464026

[ref37] Kingston A, Kirkland J and Hadjimichalis A (2020) Palliative care in non-malignant disease. *Medicine* 48(1), 37–42. doi: 10.1016/j.mpmed.2019.10.010

[ref38] Klein S, Logan A and Lindell K (2021) A scoping review of unmet needs of caregivers of patients with pulmonary fibrosis. *Current Opinion in Supportive & Palliative Care* 15(4), 226–232. doi: 10.1097/SPC.0000000000000571 34762072

[ref39] Maddocks M, Lovell N, Booth S, et al. (2017) Palliative care and management of troublesome symptoms for people with chronic obstructive pulmonary disease. *The Lancet* 390(10098), 988–1002. doi: 10.1016/S0140-6736(17)32127-X 28872031

[ref40] Mc Veigh C, Reid J, Larkin P, et al. (2017) The provision of generalist and specialist palliative care for patients with non-malignant respiratory disease in the North and Republic of Ireland: A qualitative study. *BMC Palliative Care* 17(1), 6. doi: 10.1186/s12904-017-0220-1 28693466 PMC5504568

[ref41] Mc Veigh C, Reid J, Larkin P, et al. (2018) The experience of palliative care service provision for people with non‐malignant respiratory disease and their family carers: An all‐Ireland qualitative study. *Journal of Advanced Nursing* 74(2), 383–394. doi: 10.1111/jan.13453 28910509

[ref42] Mc Veigh C, Reid J, Larkin P, et al. (2019) Palliative care for people with non-malignant respiratory disease and their carers: A review of the current evidence. *Journal of Research in Nursing* 24(6), 420–430. doi: 10.1177/1744987119840066 34394556 PMC7932269

[ref43] Molzahn A, Sheilds L, Antonio M, et al. (2021) Ten minutes to midnight: a narrative inquiry of people living with dying with advanced COPD and their family members. *International Journal of Qualitative Studies on Health and Well-Being* 16(1), 1893146–1893146. doi: 10.1080/17482631.2021.1893146 33683185 PMC7946051

[ref44] National Confidential Enquiry into Patient Outcome and Death (2024) Planning for the End. A review of the quality of care provided to adult patients towards the end of life. *Healthcare Quality Improvement Partnership*. https://www.ncepod.org.uk/2024eolc/Full%20report_end%20of%20life%20care.pdf (accessed 21 March 2025)

[ref45] Ng S Chiam Z, Chai G, et al. (2024) The PROgnostic ModEl for chronic lung disease (PRO-MEL): Development and temporal validation. *BMC Pulmonary Medicine* 24(1), 429–11. doi: 10.1186/s12890-024-03233-0. 39215286 PMC11365240

[ref46] Nicholls H, Carey M and Hambridge K (2025) What are family caregivers’ experiences of coordinating end-of-life care at home? A narrative review. *Palliative and Supportive Care* 24(23), e44, 1–13. doi: 10.1017/S1478951524001895 PMC13166439 39851071

[ref47] Noyes J, Booth A, Flemming K, et al. (2018) Cochrane qualitative and implementation methods group guidance series paper 3: Methods for assessing methodological limitations, data extraction and synthesis, and confidence in synthesized qualitative findings. *Journal of Clinical Epidemiology* 97, 49–58. doi: 10.1016/j.jclinepi.2017.06.020 29247700

[ref48] Page M McKenzie J, Bossuyt P, et al. (2021) The PRISMA 2020 statement: An updated guideline for reporting systematic reviews. *BMJ* 372, 71. doi: 10.1136/bmj.n71. PMC8005924 33782057

[ref49] Petrillo M and Bennett R (2023) *Valuing Carers 2021: England and Wales.* 1st Ed Carers UK: Centre for Care Available at: https://centreforcare.ac.uk/publications/valuing-carers-2021 (accessed 21 March 2025).

[ref50] Philip J, Gold M, Brand C, et al. (2014) Facilitating change and adaptation: The experiences of current and bereaved carers of patients with severe chronic obstructive pulmonary disease. *Journal of Palliative Medicine* 17(4), 421–427. doi: 10.1089/jpm.2013.0339 24502658

[ref51] Pinnock H, Kendall M, Murray S, et al. (2011) Living and dying with severe chronic obstructive pulmonary disease: Multi-perspective longitudinal qualitative study. *BMJ* 342, 7791. doi: 10.1136/bmj.d142 PMC3025692 21262897

[ref52] Pooler C, Richman-Eisenstat J and Kalluri M (2018) Early integrated palliative approach for idiopathic pulmonary fibrosis: A narrative study of bereaved caregivers’ experiences. *Palliative Medicine* 32(9), 1455–1464. doi: 10.1177/0269216318789025 30056786

[ref53] Rogers K, McCabe C and Dowling S (2021) What are the holistic experiences of adults living long-term with the consequences of cancer and its treatment? A qualitative evidence synthesis. *European Journal of Oncology Nursing*, 50, 101864. doi: 10.1016/j.ejon.2020.101864 33220598

[ref54] Seamark D, Blake S, Seamark C, et al. (2004) Living with severe chronic obstructive pulmonary disease (COPD): perceptions of patients and their carers: An interpretative phenomenological analysis. *Palliative Medicine* 18(7), 619–625. doi: 10.1191/0269216304pm928oa 15540670

[ref55] Spence A, Hasson F, Waldron M, et al. (2008) Active carers: Living with chronic obstructive pulmonary disease. *International Journal of Palliative Nursing* 14(8), 368–372. doi: 10.12968/ijpn.2008.14.8.30771 19023952

[ref56] Strang S, Fährn J, Strang P, et al. (2019) Support to informal caregivers of patients with severe chronic obstructive pulmonary disease: A qualitative study of caregivers’ and professionals’ experiences in Swedish hospitals. *BMJ Open* 9(8), e028720–e028720. doi: 10.1136/bmjopen-2018-028720 PMC6701698 31401598

[ref57] Strang S, Osmanovic M, Hallberg C, et al. (2018) Family caregivers’ heavy and overloaded burden in advanced chronic obstructive pulmonary disease. *Journal of Palliative Medicine* 21(12), 1768–1772. doi: 10.1089/jpm.2018.0010 30118371

[ref58] Stroebe M, Schut H and Stroebe W (2007) Health outcomes of bereavement. *The Lancet* 370(9603), 1960–1973. doi: 10.1016/S0140-6736(07)61816-9 18068517

[ref59] Tavares N, Hunt K, Jarrett N, et al. (2020) The preferences of patients with chronic obstructive pulmonary disease are to discuss palliative care plans with familiar respiratory clinicians, but to delay conversations until their condition deteriorates: A study guided by interpretative phenomenological analysis. *Palliative Medicine* 34(10), 1361–1373. doi: 10.1177/0269216320937981 32720555

[ref60] Taylor D (2017) Progressive respiratory disease: The importance of prognostic conversations and advance care planning. *Breathe* 13(4), 269–273. doi: 10.1183/20734735.012917 29209420 PMC5709798

[ref61] Thomas J and Harden A (2008) Methods for the thematic synthesis of qualitative research in systematic reviews. *BMC Medical Research Methodology* 8, 45. doi: 10.1186/1471-2288-8-45 18616818 PMC2478656

[ref62] Wilson E, Palmer J, Kaltsakas G, et al. (2025) Providing life-sustaining treatments at home for those with motor neuron disease: A qualitative study of bereaved family members’ experiences of responsibility. *Palliative Medicine* 39(5), 584–593. doi: 10.1177/02692163251327866 40119769 PMC12033379

[ref63] World Health Organization (2024) Chronic obstructive pulmonary disease (COPD). Available at https://www.who.int/news-room/fact-sheets/detail/chronic-obstructive-pulmonary-disease-(copd) (accessed 21 March 2025).

[ref64] Zhu Y, Pei X, Chen X, et al. (2023) Family caregivers’ experiences of caring for advanced cancer patients: A qualitative systematic review and meta-synthesis. *Cancer Nursing* 46(4), 270–283. doi: 10.1097/NCC.0000000000001104 35482525

