# Supplementary material for: The roles and experiences of informal caregivers in non-malignant respiratory disease at the end of life: A thematic synthesis of qualitative studies
Source: Palliat Support Care. 2026 Feb 13;24:e59. doi: 10.1017/S1478951526101643 (PMC13166666; doi:10.1017/S1478951526101643)
Supplement: Rogers et al. supplementary material 1 — Rogers et al. supplementary material [file S1478951526101643sup001.docx]

Supplementary Materials 1

Research question formation using SPIDER framework (Cooke Smith and Booth 2012),

| Sample | Informal caregivers for people with NMRD / people receiving informal care who have NMRD  (The term “caregiver” will be used to refer to a family member or friend who provides unpaid, informal support to a person with NMRD.) |
| --- | --- |
| Phenomenon of Interest | Caregiving in relation to EoL care in NMRD |
| Design | Qualitative methods |
| Evaluation | The roles and experiences of caregivers in EoL in NMRD |
| Research type | Qualitative or mixed methods |
