# Supplementary material for: The roles and experiences of informal caregivers in non-malignant respiratory disease at the end of life: A thematic synthesis of qualitative studies
Source: Palliat Support Care. 2026 Feb 13;24:e59. doi: 10.1017/S1478951526101643 (PMC13166666; doi:10.1017/S1478951526101643)
Supplement: Rogers et al. supplementary material 2 — Rogers et al. supplementary material [file S1478951526101643sup002.docx]

Quality Assessment using JBI Critical Appraisal Checklist for Qualitative Research (2020)

1. Is there congruity between the stated philosophical perspective and the research methodology?

2. Is there congruity between the research methodology and the research question or objectives?

3. Is there congruity between the research methodology and the methods used to collect data?

4. Is there congruity between the research methodology and the representation and analysis of data?

5. Is there congruity between the research methodology and the interpretation of results?

6. Is there a statement locating the researcher culturally or theoretically?

7. Is the influence of the researcher on the research, and vice- versa, addressed?

8. Are participants, and their voices, adequately represented?

9. Is the research ethical according to current criteria or, for recent studies, and is there evidence of ethical approval by an appropriate body?

10. Do the conclusions drawn in the research report flow from the analysis, or interpretation, of the data?

| Studies | Q.1 | q.2 | Q.3 | Q.4 | Q.5 | Q.6 | Q.7 | Q.8 | Q.9 | Q.10 |
| --- | --- | --- | --- | --- | --- | --- | --- | --- | --- | --- |
| Bajwah, Koffman et al. (2013) | Unclear | Yes | Yes | Yes | Yes | No | No | Yes | Yes | Yes |
| Bajwah, Higginson et al. (2013) | Unclear | Yes | Yes | Yes | Yes | No | No | Yes | Yes | Yes |
| Brown et al. (2012) | Unclear | Yes | Yes | Yes | Yes | No | No | Yes | Yes | Yes |
| Cawley et al. (2014) | Unclear | Yes | Yes | Yes | Yes | No | No | Yes | Yes | Yes |
| Egerod et al. (2019) | Unclear | Yes | Yes | Yes | Yes | No | No | Yes | Yes | Yes |
| Ek et al. (2011) | Unclear | Yes | Yes | Yes | Yes | No | No | Yes | Yes | Yes |
| Ek et al. (2015) | Yes | Yes | Yes | Yes | Yes | Yes | No | Yes | Yes | Yes |
| Elkington et al. (2004) | Unclear | Yes | Yes | Yes | Yes | No | No | Yes | Yes | Yes |
| Ferreira et al. (2020) | Unclear | Yes | Yes | Yes | Yes | Yes | No | Yes | Yes | Yes |
| Fusi-Schmidhauser et al. (2020) | Unclear | Yes | Yes | Yes | Yes | No | No | Yes | Yes | Yes |
| Guthrie et al. (2001) | Unclear | Yes | Yes | Yes | Yes | No | No | Yes | No |  |
| Hasson et al. (2008) | Unclear | Yes | Yes | Yes | Yes | No | Unclear | Yes | Yes | Yes |
| Hasson et al. (2009) | Unclear | Yes | yes | yes | Yes | No | Unclear | Yes | yes | Yes |
| Kalluri et al. (2022) | Yes | Yes | Yes | Yes | Yes | No | Unclear | Yes | Yes | Yes |
| Mc Veigh et al. (2017) | Yes | Yes | Yes | Yes | Yes | Yes | Unclear | Yes | Yes | Yes |
| Molzahn et al. (2021) | Unclear | Yes | Yes | Yes | Yes | No | Unclear | Yes | Yes | Yes |
| Philip et al. (2014) | Unclear | Yes | Yes | Yes | Yes | No | Unclear | Yes | Yes | Yes |
| Pooler et al. (2018) | Yes | Yes | Yes | Yes | Yes | No | Unclear | Yes | Yes | Yes |
| Seamark et al. (2004) | Unclear | Yes | Yes | Yes | Yes | No | No | Yes | Yes | Yes |
| Spence at al. (2008) | Unclear | Yes | Yes | Yes | Yes | Yes | Yes | Yes | Yes | Yes |
| Strang et al. (2018) | Unclear | Yes | Yes | Yes | Yes | No | No | Yes | Yes | Yes |
